# Impact of normalized COVID-19 prevention and control measures on lower respiratory tract infection pathogenesis in hospitalized children

**DOI:** 10.3389/fpubh.2024.1367614

**Published:** 2024-02-27

**Authors:** Yuan Feng, Huaixiao Zhang, Bo Zhang, Yinfei Zhou, Haibin Yuan

**Affiliations:** Department of Pediatrics, Xiangtan Central Hospital, Xiangtan, China

**Keywords:** COVID-19 pandemic, respiratory pathogens, epidemiological characteristics, children, lower respiratory tract infection

## Abstract

**Objective:**

This study aimed to investigate the epidemiological characteristics of common pathogens contributing to childhood lower respiratory tract infections (LRTIs) in Xiangtan City, Hunan Province before and during the coronavirus disease 2019 (COVID-19) pandemic.

**Methods:**

A total of 11,891 enrolled patients, aged 1 month to 14 years, diagnosed with LRTIs and admitted to Xiangtan Central Hospital from January 2018 to December 2021 were retrospectively reviewed in this study. Specifically, the epidemiological characteristics of these pathogens before and during the COVID-19 pandemic were analyzed.

**Results:**

There was a significant decrease in the number of children hospitalized with LRTIs during the COVID-19 pandemic (2020–2021) compared to data from 2018 to 2019 (before the COVID-19 pandemic). Of these cases, 60.01% (7,136/11,891) were male and 39.99% (4,755/11,891) were female. 78.9% (9,381/11,891) cases occurred in children under 4 years of age. The average pathogen detection rate among 11,891 hospitalized LRTIs children was 62.19% (7,395/11,891), with the average pathogen detection rate of 60.33% (4,635/7,682) and 65.57% (2,670/4,209) before and during COVID-19 pandemic, respectively. The detection rates of adenovirus (ADV), *bordetella pertussis* (BP) and *moraxella catarrhalis* (*M. catarrhalis*) decreased dramatically, while the detection rates of influenza viruses (IFV), parainfluenza viruses (PIV), respiratory syncytial virus (RSV), *haemophilus influenzae* (*H. influenzae*), *streptococcus pneumoniae* (*S. pneumoniae*), and *staphylococcus aureus* (*S. aureus*) increased significantly during the COVID-19 pandemic. Overall, RSV, *mycoplasma pneumoniae* (MP), *H. influenzae*, and IFV were the major pathogens causing LRTIs in hospitalized children before and during the COVID-19 pandemic.

**Conclusion:**

Public health interventions for COVID-19 prevention are beneficial to reduce the incidence of LRTIs in children by limiting the prevalence of ADV, MP, BP, and *M. catarrhalis*, but which have limited restrictive effects on other common LRTIs-associated pathogens. Collectively, the data in this study comprehensively investigated the effects of COVID-19 pandemic on the epidemiological characteristics of respiratory pathogens, which will be beneficial for improving early preventive measures.

## 1 Introduction

Lower respiratory tract infections (LRTIs) are responsible for a high level of morbidity and mortality in humans, which have been considered one of the most significant factors threatening public health worldwide (1). The rapid spread of LRTIs-related pathogens within a short time are frequently observed in some specific occasions (e.g., kindergarten and playground), owing to their high contagion (2). The outbreak or occurrence of LRTIs can be caused by a variety of pathogens, including viruses, bacteria, and *mycoplasma pneumoniae* (MP), while the epidemiological characteristics of these infectious agents vary from regions, seasons, and other factors (3). Thus, updating the information on the epidemiological features of LRTIs-related pathogens is essential for the early diagnosis and treatment of this disease.

Since the end of December 2019, a novel human coronavirus, called the severe acute respiratory syndrome coronavirus 2 (SARS-CoV-2), emerged in Wuhan city, China (4). Subsequently, the disease (coronavirus disease 2019, COVID-19) caused by SARS-CoV-2 has been documented in all provinces or regions in China, the threats of which to public health received widespread concern (5). In view of these, a variety of strict non-pharmaceutical interventions (NPIs) measures were performed to limit the spread of SARS-CoV-2 nationwide, and which effectively restricted the prevalence of this infectious virus and saved the lives of thousands of people in China (6).

During the COVID-19 pandemic in Shanghai city of China in 2020, the decreased detection rates of Human rhinovirus (HRV), Human parainfluenza virus (HPIV), *Haemophilus influenzae* (*H. influenzae*), and MP, and the increased detection rates of Influenza B virus (FluB) and most of tested bacteria [including *Staphylococcus aureus* (*S. aureus*), *Escherichia coli* (*E. coli*), and *Klebsilla pneumoniae* (*K. pneumoniae*)] were observed compared with these in 2019 (7). In western China (Gansu, Qinghai, Xinjiang, and Inner Mongolia), the positive rates of influenza virus (IFV), and *Streptococcus pneumoniae* (*S. pneumoniae*) among patients with acute respiratory infections decreased, but other types of viruses and bacteria showed higher prevalence tendency during the COVID-19 pandemic (8). In Henan province of China, the positive detection rates of IFV, and human metapneumovirus (HMPV), and human respiratory syncytial virus (HRSV) decreased sharply, and the detection rates of HRV and human bocavirus significantly increased (9). These data suggested that NPIs have broad effects on the transmission of these respiratory pathogens, while the changes of different respiratory pathogens before and during COVID-19 pandemic varied in different regions (10, 11).

This study conducted a retrospective study from 2018 to 2021 to analyze the epidemiological characteristics of pathogen-associated LRTIs among inpatients (aged 1 month to 14 years) in Xiangtan Central Hospital, Hunan Province, China. In particular, the impact of the COVID-19 pandemic on the number of LRTIs-positive patients, the type of pathogens and their epidemiological characteristics were studied. The results will provide references for clinical diagnosis and treatment, and to help formulate strategies for the prevention and control of respiratory infections in children in the public health sector.

## 2 Methods

### 2.1 Study design

From January 2018 to December 2021, all hospitalized children from Xiangtan Central Hospital diagnosed with LRTIs were included in this retrospective study. This was a retrospective study that followed ethical standards, obtained informed consent from the children’s families, and was approved by the Ethics Committee of Xiangtan Central Hospital (NO. 2023-KC-58-09-019).

Inclusion criteria: (1) Meet the diagnostic criteria for LRTIs according to the 8th edition of Zhufutang Practical Paediatrics (12); (2) Age from 1 month to 14 years; (3) The clinical samples from inpatients were collected for pathogen detection.

Exclusion criteria: (1) Presence of congenital airway malformations such as esophageal tracheal atresia and congenital tracheal chondrodysplasia; (2) Presence of intracranial infections, congenital immunodeficiencies, leukemias, and other systemic disorders as primary diagnosis; (3) Acute exacerbation of bronchial asthma with normal chest imaging.

### 2.2 Specimen collection

Nasal or throat swabs from enrolled patients were collected individually at the collection site within 24 h and then sent to the clinical laboratory center for antigenic or nucleic acid testing for common pathogens, including influenza A and B viruses (FluA and FluB), adenovirus (ADV), HPIV 1–3, HRSV, *Bordetella pertussis* (BP), and MP. Sputum samples were also collected for bacterial cloning and molecular characterization.

### 2.3 Pathogens detection

A multiplex direct immunofluorescence assay kit (Diagnostic Hybrids, Inc., United States and Bierce Spain Ltd.) was used to simultaneously detect the fluorescent antigens of RSV, ADV, PIV-1, PIV-2, PIV-3, Flu A, and Flu B. Using the commercially available reagent kits to individually extract total nucleic acid from collected samples and perform individual pathogen nucleic acid testing (Shenzhen Yicubic Biotechnology Co., Guangzhou Da’an Gene Co., Shengxiang Biotechnology Co., China).

The sputum samples were mixed with sterilized normal saline. The supernatants were then collected and plated on Columbia agar plates containing 5% defibrinated sheep blood. After 24 h of incubation at 37°C and 5% CO2, individual colonies on the plate were selected and purified. Finally, the 16S rRNA gene of the purified isolates was amplified by PCR using primers 27F 5′-AGAGTTTGATCCTGGCTCAG-3′ and 1492R 5′-TACCTTGTTACGACTT-3′ according to a recently published research (13).

### 2.4 Clinical data capture and management

Clinical data of children hospitalized with LRTIs from January 2018 to December 2021 were retrospectively analyzed. Data were retrieved from our clinical case database, which collected basic information about children, including sex, age, admission time, discharge diagnosis (including bronchiolitis and community-acquired pneumonia), respiratory pathogen test results, etc. Two physicians independently validated the data and resolved any discrepancies through re-export and subsequent review, following a meticulous process to ensure data accuracy. A total of 11,891 cases were finally included in this analysis.

### 2.5 Statistical analysis

The data obtained were analyzed using SPSS 26.0 software, and count data were expressed as percentages (%), and comparisons between groups were made using the chi-squared test, *Post-hoc* pairwise comparisons were performed with partitions of the χ^2^ method, with *p* < 0.05 was considered to be statistically different and the adjusted test level was α’ = 0.0083.

## 3 Results

### 3.1 Study population

**Figure 1:**
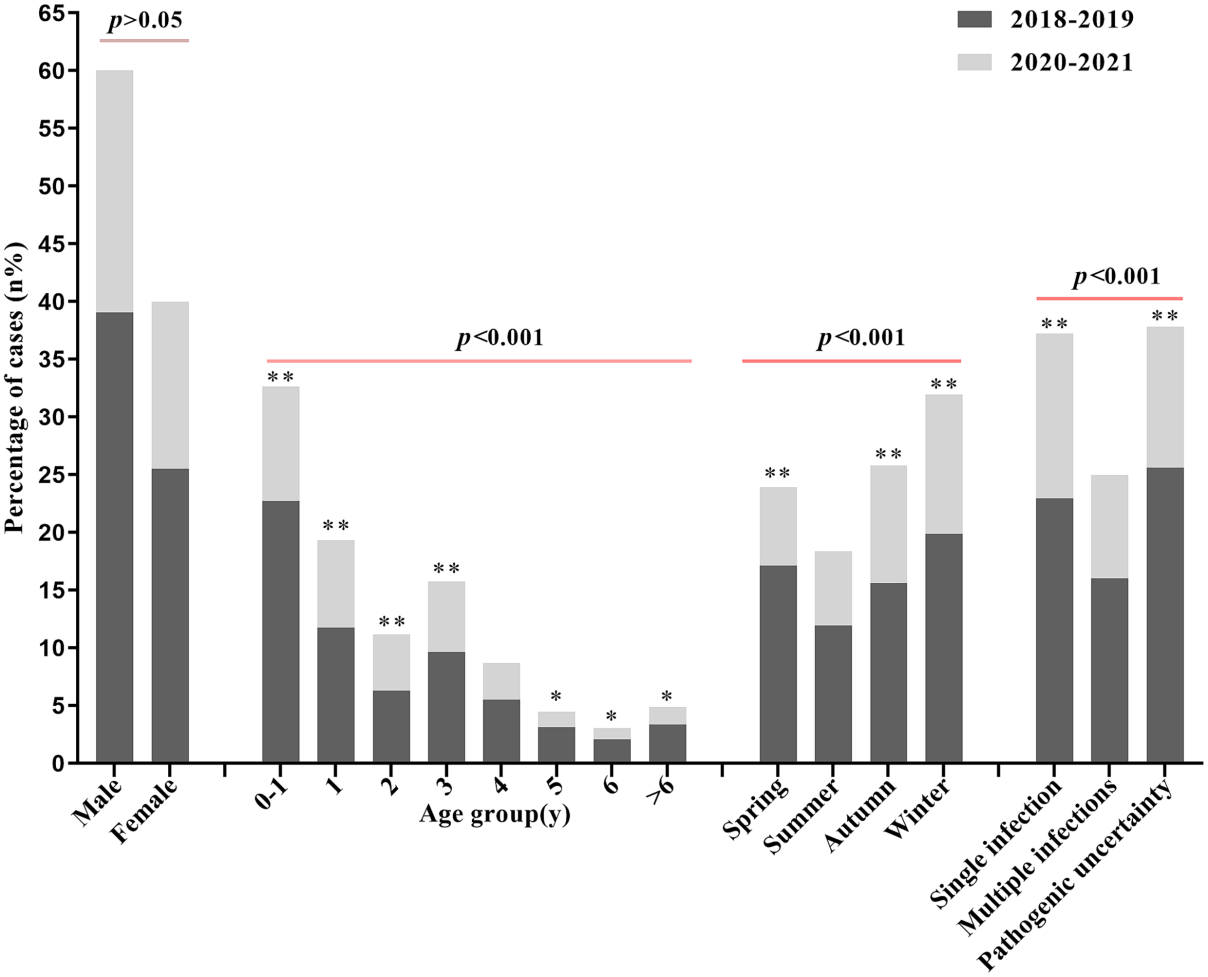
Percentage comparison of the number of LRTIs in hospitalized children by gender, age, season, and etiologic diagnosis in two different time periods. * indicates *p* < 0.05 and ** indicates *p* < 0.01 for comparison between the two groups (2018–2019, 2020–2021).

A total of 11,891 hospitalized children diagnosed as LRTIs during 2018–2021 were included in this research, among which 7,136 (60.01%) cases were male and 4,755 (39.99%) cases were female. Considering the initial report of COVID-19 disease in the end of 2019 in China, we divided the collected samples into two groups based on the sampling time: 7,682 (64.60%) cases from 2018 to 2019 and 4,209 (35.40%) cases from 2020 to 2021. According to the patients’ age, the children were divided into four groups, as follows: 0 ~ 1 year (3,882, 32.65%), 1 years (2,300, 19.34%), 2 years (1,327, 11.16%), 3 years (1,872, 15.74%), 4 years (1,033, 8.69%), 5 years (534, 4.49%), 6 years (360, 3.03%) and 6 ~ 14 years (583, 4.90%). In addition, the cases in spring, summer, autumn, and winter during 2018–2021 were 2,844 (23.92%), 2,182 (18.35%), 3,068 (25.80%), and 3,797 (31.93%), respectively. In contrast, the rate of pathogen-positive tests for children hospitalized in 2018–2019 was lower than in 2020–2021, with significant differences by age 3 years and in winter and spring at two different times (*p* < 0.05) (Table 1). However, the number of LRTIs cases by age, gender, and season was significantly higher in 2018–2019 than in 2020–2021, but there was no significant difference in the proportion of child gender, summer, and multiple infections between the two periods (*p* > 0.05) (Table 2; Figure 1).

**Table 1:** Demographic characteristics and pathogen detection profile of LRTI in hospitalized children before and during the COVID-19 pandemic (2018–2021).

Characteristics	Total (*n*, %)	2018–2019 (*n*, %)	2020–2021 (*n*, %)	χ^2^	*p*-value
	11,891 (7,395, 62.19%)	7,682 (4,635, 60.33%)	4,209 (2,760, 65.57%)	31.73	<0.001
Gender				1.95	0.163
Male	7,136 (4,474, 62.70%)	4,644 (2,832, 60.98%)	2,492 (1,642, 65.89%)	16.71	<0.001
Female	4,755 (2,921, 61.43%)	3,038 (1,803, 59.35%)	1,717 (1,118, 65.11%)	15.39	<0.001
Age				57.11	<0.001
0–1 years	3,882 (2,386, 61.46%)	2,703 (1,608, 59.49%)	1,179 (778, 65.99%)	14.64	<0.001
1 years	2,300 (1,317, 57.26%)	1,397 (731, 52.33%)	903 (586, 64.89%)	35.40	<0.001
2 years	1,327 (805, 60.66%)	753 (424, 56.31%)	574 (381, 66.38%)	13.84	<0.001
3 years	1,872 (1,199, 64.05%)	1,147 (720, 62.77%)	725 (479, 66.07%)	2.10	0.148
4 years	1,033 (689, 66.70%)	656 (442, 67.38%)	377 (247, 65.52%)	0.37	0.541
5 years	534 (353, 66.10%)	372 (250, 67.20%)	162 (103, 63.58%)	0.66	0.416
6 years	360 (247, 68.61%)	251 (175, 69.72%)	109 (72, 66.10%)	0.47	0.491
>6 years	583 (399, 68.44%)	403 (285, 70.72%)	180 (114, 63.33%)	3.14	0.076
Season				28.26	<0.001
Spring (Mar-May)	2,844 (1,784, 62.73%)	2,037 (1,236, 60.67%)	807 (548, 67.91%)	12.92	<0.001
Summer (Jun-Aug)	2,182 (1,350, 61.87%)	1,421 (852, 59.58%)	761 (498, 65.44%)	6.31	0.012
Autumn (Sep-Nov)	3,068 (1,799, 58.64%)	1,860 (1,083, 58.23%)	1,208 (716, 59.27%)	0.33	0.566
Winter (Jan-Feb, Dec)	3,797 (2,462, 64.84%)	2,364 (1,464, 61.93%)	1,433 (998, 69.64%)	23.29	<0.001

**Table 2:** Detection rates of various pathogens in hospitalized children with LRTI before and during the COVID-19 pandemic (2018–2021).

Characteristics	Total	2018–2019	2020–2021	χ^2^	*p*-value
	*n* = 11,891	*n* = 7,682	*n* = 4,209		
**Diagnosis**					
Single infection	4,425 (37.21%)	2,728 (35.51%)	1,697 (43.41%)	21.78	<0.001
Multiple infections	2,970 (24.98%)	1,907 (24.82%)	1,063 (27.19%)	0.27	0.604
Pathogenic uncertainty	4,496 (37.81%)	3,047 (39.67%)	1,449 (37.07%)	31.73	<0.001
**Pathogen**					
Viral	3,118 (26.22%)	1,631 (21.23%)	1,487 (35.33%)	279.34	<0.001
FluA	435 (3.65%)	290 (3.78%)	145 (3.44%)	0.84	0.359
FluB	464 (3.90%)	177 (2.30%)	287 (6.82%)	147.80	<0.001
PIV1	82 (0.69%)	33 (0.43%)	49 (1.16%)	21.43	<0.001
PIV2	40 (0.34%)	8 (0.10%)	32 (0.76%)	34.92	<0.001
PIV3	237 (1.99%)	63 (0.82%)	174 (4.13%)	152.87	<0.001
ADV	658 (5.53%)	536 (6.98%)	122 (2.90%)	86.54	<0.001
RSV	1,202 (10.11%)	524 (6.82%)	678 (16.11%)	272.66	<0.001
*Mycoplasma pneumoniae*	1,552 (13.05%)	1,034 (13.46%)	518 (12.31%)	3.19	0.074
Bacterial	3,654 (30.73%)	2,256 (29.38%)	1,398 (33.21%)	18.91	<0.001
*H. influenzae*	1,153 (9.70%)	637 (8.29%)	516 (12.26%)	48.88	<0.001
*S. aureus*	398 (3.35%)	237 (3.09%)	161 (3.83%)	4.60	0.032
*S. pneumoniae*	536 (4.51%)	304 (3.96%)	232 (5.51%)	15.27	<0.01
*K. pneumoniae*	308 (2.59%)	193 (2.51%)	115 (2.73%)	0.52	0.470
*M. catarrhalis*	404 (3.40%)	325 (4.23%)	79 (1.88%)	45.90	<0.001
*P. aeruginosa*	222 (1.87%)	117 (1.52%)	105 (2.49%)	14.70	<0.001
*E. coli*	458 (3.85%)	285 (3.71%)	173 (4.11%)	1.18	0.278
*B. pertussis*	175 (1.47%)	158 (2.06%)	17 (0.40%)	51.23	<0.001

### 3.2 Overall detection of LRTIs-associated pathogens

Of 11,891 tested samples/patients, 62.19% (7,395/11,891) of LRTIs-patients were diagnosed with at least one respiratory pathogen. Among 7,395 cases, 4,425 (59.84%) cases had single pathogen infection, 2,970 cases (40.16%) had two or more than two pathogen infection. According to pathogen characteristics, bacterial infection (30.73%, 3,654/11,891) was the most common factors resulting in LRTIs, followed by viral infection (26.22%, 3,118/11,891), and MP infection (13.05%, 1,552/11,891). However, the number of cases documented in 2018–2019 (*n* = 7,682) was much higher that these during CDVID-19 pandemic (*n* = 4,209), the detection rates of these pathogens greatly changed during these two periods. By comparing infections with LRTIs-associated pathogens before and during the COVID-19 pandemic, we found that the case number of FluA, ADV, MP, and bacterial infection significantly decreased during COVID-19 pandemic. Analysis of positive LRTIs pathogen test results showed only the prevalence of ADV, BP, and *M. catarrhalis* remarkably decreased during COVID-19 pandemic, while the detection rates of FluB, PIV, RSV, *H. influenzae*, *S. pneumoniae*, and *P. aeruginosa* significantly increased (Table 2).

### 3.3 Influence of age on LRTIs incidence

**Figure 2:**
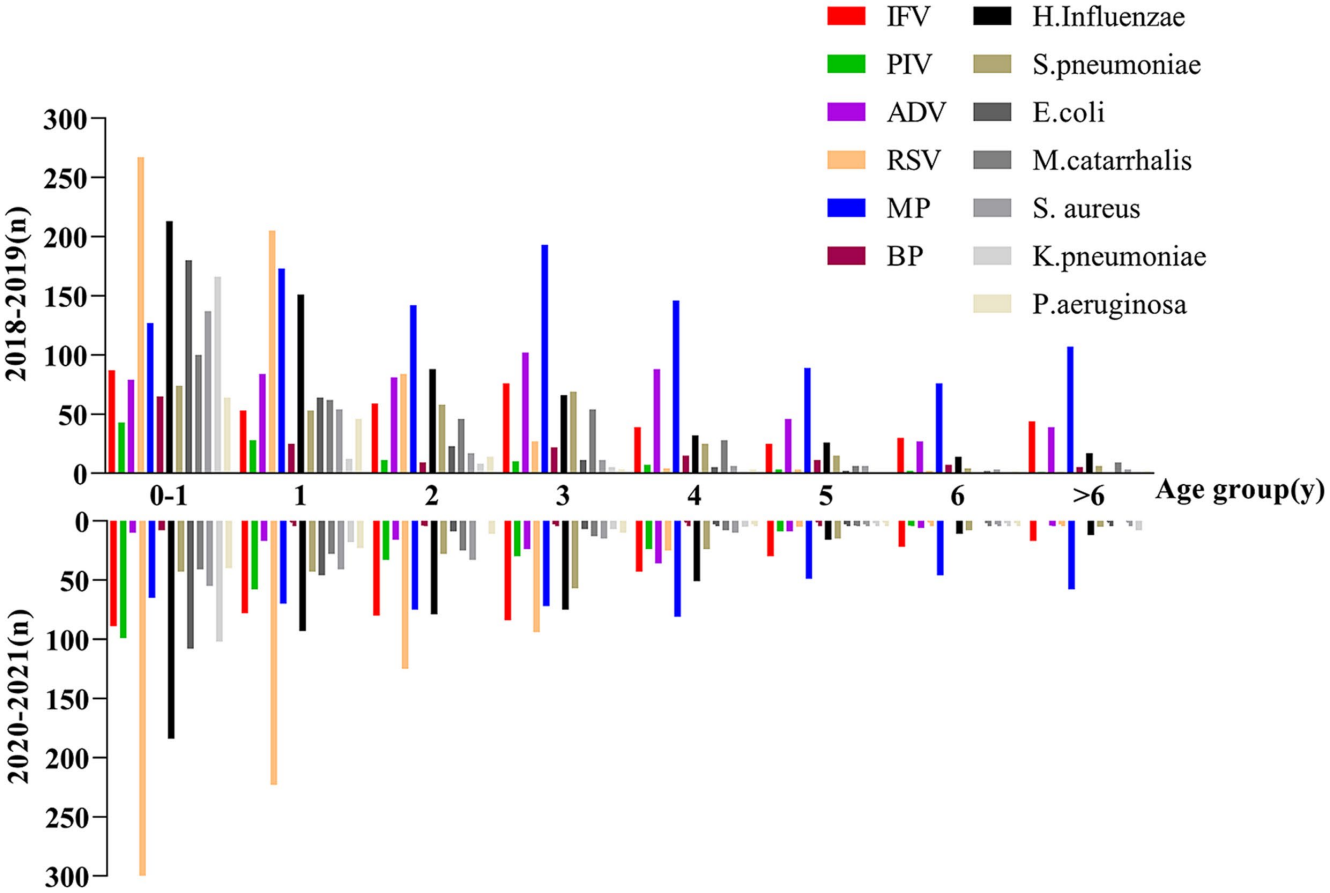
Comparison of detection of different pathogens in hospitalized children with LRTIs in different age groups before and during the COVID-19 pandemic.

To further characterize the relationship between age and pathogen infection, data were stratified by age. There was a more statistically significant difference between infants and toddlers (0–2 years), who had a higher relative rate of pathogen-positive detection during the COVID-19 pandemic. In contrast, there was no statistically significant difference between the two groups in pathogen-positive detection rates among children aged 3 years and older (Table 1). Among these, RSV and bacterial infections were the most common causes of LRTIs in infants under 4 years of age, and RSV was also the predominant epidemiological pathogen in infants and children after normative control of COVID-19 pandemic, whereas the prevalence of MP and IFV remained relatively stable in all age groups, and LRTIs in school-aged children was predominantly characterized by MP, IFV, and ADV (Table 2; Figure 2). Thus, during the COVID-19 pandemic, the pathogens of respiratory infections in children shifted to a viral spectrum, with the age of infection progressively favoring younger children, especially those under 2 years of age.

### 3.4 Influence of seasonal changes on the prevalence of respiratory pathogens

**Figure 3:**
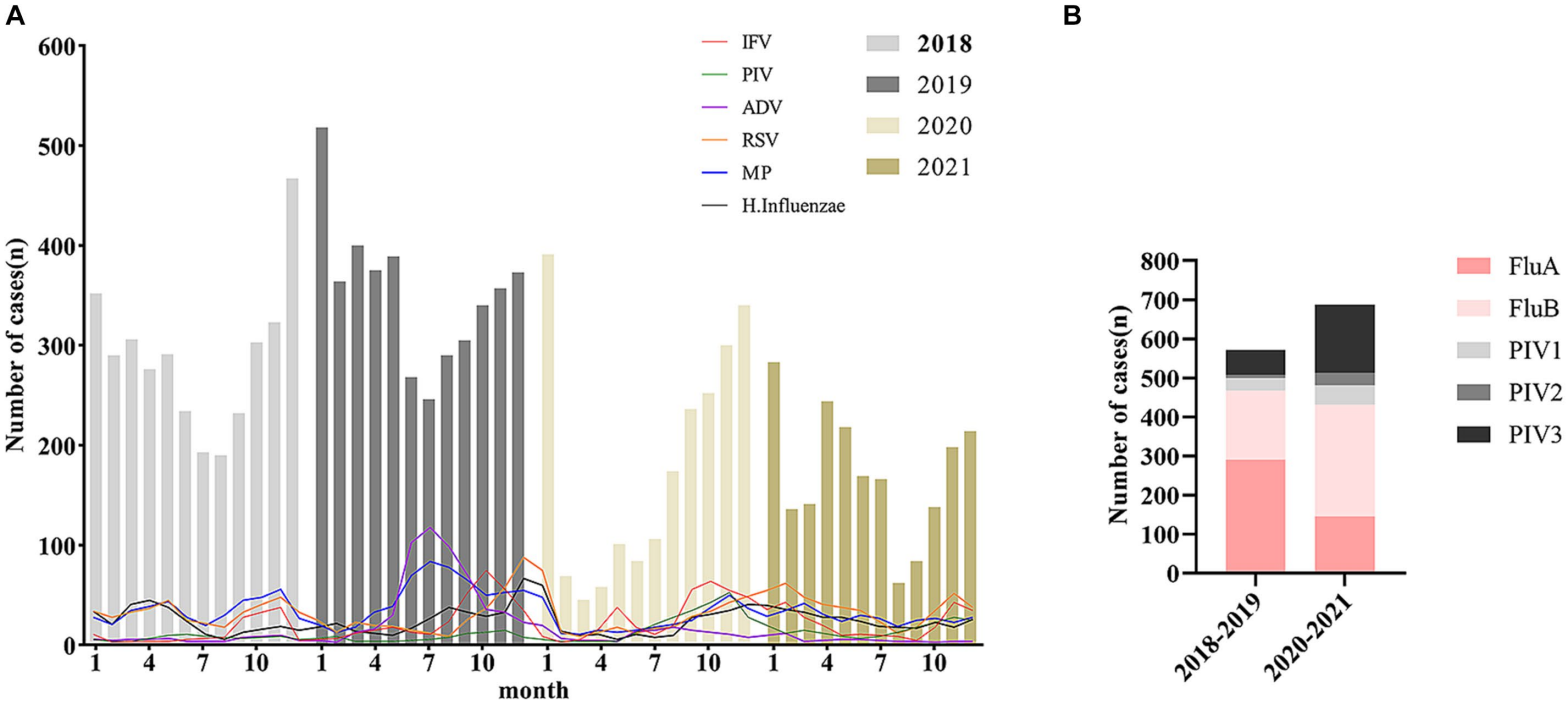
Seasonal epidemiological trends of common viruses and *Haemophilus influenzae* in pediatric LRTIs **(A)**. Detection of influenza and parainfluenza virus subtypes in children with LRTIs before and during the COVID-19 pandemic **(B)**.

The overall prevalence of LRTIs in hospitalized children in the four seasons was lowest in summer, highest in winter, and intermediate in spring and autumn, while the rates of positive tests were spring (1,784/2,844, 62.73%), summer (1,350/2,182, 61.87%), autumn (1,799/3,068, 58.64%), and winter (2,462/3,797, 64.84%), respectively. The data results showed a decrease in the prevalence of LRTIs in children in the spring before and during the COVID-19 pandemic, no significant difference in prevalence between the summer months, and a relative increase in prevalence in the autumn and winter months (Figure 1), but the rate of pathogen-positive tests increased except in the autumn, and the difference was statistically significant (Table 1). We again analyzed the seasonal epidemiological characteristics of the different pathogens and found that RSV and MP had higher detection rates in the perennial year compared with other pathogens, while ADV had an outbreak in the summer of 2019 (Figure 3A), and there was a general increase in the rate of positive tests for IFV in the winter, whereas the positive rates of FluB and PIV increased significantly in 2020–2021 (Table 2; Figure 3B).

## 4 Discussion

COVID-19 pandemic has inflicted significant global human and economic losses (5). The main modes of respiratory pathogen transmission are droplet and contact, since the initial outbreak of COVID-19 in late 2019 (14), China has implemented a series of pandemic prevention and control measures (15). Strict NPIs, including social distancing and wearing masks, were beneficial in minimizing the spread of respiratory pathogens in both adult and pediatric populations. These measures have not only effectively contained the large-scale spread of COVID-19, but have also contributed to a reduction in infections and hospitalizations related to common respiratory pathogens in children in China. As a result, the epidemiological characteristics of childhood LRTIs have been affected and the spectrum of common pathogens has changed (16–19). Notably, the incidence of LRTIs was significantly reduced after the COVID-19 outbreak, suggesting the effectiveness of COVID-19 prevention and control measures in reducing childhood respiratory infections (16, 19). Detection rates for different pathogens show significantly variation with age, with the highest rates, particularly for viral infections (17–21), occurring before the age of 3 years, this is attributed to lower immunity and increased environmental contact in young children, coupled with challenges in adhering to preventive measures liking handwashing and mask-wearing (18, 19). While the incidence rate of LRTI usually decreases with age, the incidence rate of LRTI increases significantly at the age of 3 years, which may be due to the fact that children begin to learn in early childhood settings at this age (22). The closure of schools during the COVID-19 pandemic, a crucial measure to reduce transmission, particularly in schools, significantly lowered cross-infections (15).

Firstly, our study indicated that the infection rate of LRTIs was observed to be higher in boys than in girls (Table 1; Figure 1), a trend possibly linked to the demographic composition of children in Xiangtan. This finding aligns with domestic investigations (23, 24). Compared to 2018–2019, the incidence of LRTIs decreased significantly in 2020–2021, but pathogen positivity and single pathogen positivity increased over the same period, this observation may be attributed to the preventive measures implemented for COVID-19 pandemic (Table 2). Before and during the COVID-19 pandemic, there was no statistically significant difference in the incidence of LRTIs among children in summer (Figure 1), but there was no statistically significant difference in the rate of pathogen positivity compared with that in autumn (Table 1), and the difference in seasonal incidence was attributed to the change in temperature in autumn and winter, which is favorable for pathogen multiplication, and the peak of pathogen infection started in autumn (25). The increase in the pathogen-positive detection rate may be due to the systematic training of all sampling personnel in health care facilities on respiratory specimen collection during the COVID-19 pandemic, and the prevention and control measures that allowed children to attend the hospital relatively late (17).

Secondly, our study identified the three most common pathogens for LRTIs in children as RSV, MP, and *H. influenzae* (Table 2; Figure 2). Overall detection rates for common viral infections were significantly different before and during the COVID-19 pandemic, and the outbreak of ADV infections in the summer of 2019 was contained during the COVID-19 pandemic, but the rate of positive tests for IFV, PIV, and RSV was elevated due to the fact that ADV predominantly infects children over 3 years of age and correlates with average temperatures and humidity during the ADV season (25, 26). Although the overall LRTIs positive rate decreased during routine COVID-19 prevention and control activities. However, viral prevalence actually increased in relative terms. The RSV pandemic remained prevalent, while IFV and PIV showed some outbreaks in autumn or winter (Figure 3A), with variations in different subtypes (Figure 3B), these results are inconsistent other studies (16–19). Post-COVID-19 prevention and control, the overall incidence of LRTIs decreased, but the prevalence of MP and IFV remained relatively stable across all age groups (Figure 2).

Finally, we found that there were outbreaks of ADV, IFV, RSV, and MP infections in children with LRTIs in 2019, the prevention and control of COVID-19 have also controlled the outbreak and infection of various pathogens (Figure 3). However, during the COVID-19 pandemic, MP and RSV were common pathogens causing LRTIs in hospitalized children, while IFV and PIV continued to outbreak during the autumn and winter seasons, thus requiring continued attention. While bacterial infections in LRTIs in infants and hospitalized children are often secondary to viral infections, some pathogens (e.g., *H. influenzae* and *S. pneumoniae*) also show a relatively high rate of positive detection, making viral and bacterial infections the main causes of LRTIs in children under 3 years of age (27). Therefore, we must continue to emphasize the importance of prevention and control of viral and bacterial respiratory infections in infants and young children, and pay more attention to MP, IFV, and ADV infections in school-aged children. It emphasizes the need for focused preventive measures in early childhood centers or schools where younger children engage in group learning, potentially increasing the risk of cross-infections. Measures such as environmental disinfection and indoor air circulation should be reinforced in these settings.

Overall, pathogenetic studies conducted before and after COVID-19 not only contribute to a more accurate medical basis for clinical diagnosis and treatment, and evidence-based medicine, but also enhance healthcare professionals’ comprehension of regional respiratory infection characteristics caused by common pathogens. The study acknowledges limitations such as not testing for other common or rare respiratory pathogens in children such as HRV, Chlamydia and HMPV, and not testing for different subtypes of common viruses, thus not reflecting the epidemiological status of these pathogens (20–26). To enhance the surveillance of respiratory pathogens in children, more comprehensive multiplex assays are needed to monitor LRTIs for common respiratory pathogens as well as infections with different viral subtypes. Consequently, vigilance against potential pandemics of rare pathogens such as HMPV is crucial in addition to preventing and controlling outbreaks of common childhood respiratory infections and MP. Targeted measures to prevent and control pandemic outbreaks of common respiratory pathogens in children are essential, with particular emphasis on the persistent challenge of preventing and controlling RSV. This remains a major concern and an urgent priority in the current landscape.

## 5 Conclusion

Our results suggest that standardized prevention and control measures implemented during the COVID-19 pandemic reduced the incidence of LRTIs in children, while also influencing the pathogen profile of LRTIs in hospitalized children. Overall, this impact has resulted in a reduction in outbreaks of common pathogens and more infectious pathogens while tilting toward viral infections and affecting younger age groups. Therefore, attention needs to be paid to potential outbreaks and pandemics of common pathogens, especially viral respiratory infections in infants and young children. In addition, after the relaxation of pandemic-related measures, continuous surveillance and targeted prevention and control efforts are essential to manage and mitigate the evolving situation of respiratory infections in children.

## Data availability statement

The raw data supporting the conclusions of this article will be made available by the authors, without undue reservation.

## Ethics statement

The studies involving humans were approved by Ethics Committee of Xiangtan Central Hospital. The studies were conducted in accordance with the local legislation and institutional requirements. Written informed consent for participation in this study was provided by the participants’ legal guardians/next of kin.

## Author contributions

YF: Funding acquisition, Investigation, Software, Writing – original draft, Writing – review & editing. HZ: Data curation, Writing – original draft. BZ: Data curation, Writing – original draft. YZ: Data curation, Funding acquisition, Validation, Writing – review & editing. HY: Supervision, Visualization, Writing – review & editing.
